# Purpura Hemorrhagica

**Published:** 1902-07

**Authors:** C. A. Paisley

**Affiliations:** Winfield, Iowa


### PURPURA HEMORRHAGICA.

By C. A. Paisley, V.S.,

WINFIELD, IOWA.

On June 1, 1900, I was called to see a mare, six years of age, exhibiting the characteristic symptoms of epizooty. General treatment was administered, with apparently satisfactory results; but on the evening of the 7th the patient began to show much uneasiness, with difficult breathing. The next morning the lips, limbs, breast, and abdomen were much swollen, and the general surface was rough with vesicles; petechial spots of a purplish hue were numerous on the mucous membrane, particularly of the nostrils; temperature much elevated; bowels constipated; urine highly colored; standing, and disinclined to move.

Potassium chlorate had been given every few hours since the previous evening until 2 P.M., when 30 c.c. of Pasteur's antistreptococcus serum was injected. The pulse at this time was 96; temperature, 105½° F. At 8.30 P.M. temperature was 105¾°; pulse, 96; the serum was again injected.

*June 9th.* Temperature, 103°; pulse, 80 in the morning; at noon, temperature was 103½°; pulse, 60.

10*th.* Morning: temperature, 105½°; pulse, 60. At noon, temperature was 104½°; pulse, 52.

Patient became convalescent until about the 22d of the month, when the petechial spots reappeared within the nostrils. The prompt administration of serum arrested the further development of untoward symptoms, and convalescence ensued with ultimate recovery.

During the course of treatment 130 c.c. of antistreptococcus serum was administered, the greater portion being used during the earlier period of the attack. Potassium chlorate and tonics were continued to the close of July.

Cracks and fissures appeared at the flexures of the hind limbs, from which there was a slight amber-colored discharge.

There was great debility, with almost complete loss of appetite in the earlier period of the disease.

